# New Horizons in Myotonic Dystrophy Type 1: Cellular Senescence as a Therapeutic Target

**DOI:** 10.1002/bies.202400216

**Published:** 2024-12-26

**Authors:** Cécilia Légaré, J. Andrew Berglund, Elise Duchesne, Nicolas A. Dumont

**Affiliations:** ^1^ RNA Institute College of Arts and Sciences University at Albany‐SUNY Albany New York USA; ^2^ School of Rehabilitation Sciences Faculty of Medicine Université Laval Quebec Quebec Canada; ^3^ CHU de Québec – Université Laval Research Center Québec Québec Canada; ^4^ Groupe de Recherche Interdisciplinaire sur les Maladies Neuromusculaires (GRIMN) Centre intégré universitaire de santé et de services sociaux du Saguenay‐Lac‐Saint‐Jean Saguenay Quebec Canada; ^5^ Department of Biological Sciences, College of Arts and Sciences University at Albany‐SUNY Albany New York USA; ^6^ Centre Interdisciplinaire de Recherche en Réadaptation et Intégration Sociale (Cirris) Centre Intégré Universitaire de Santé et de Services Sociaux Capitale‐Nationale Québec Quebec Canada; ^7^ CHU Sainte‐Justine Research Center Montreal Quebec Canada; ^8^ School of rehabilitation Faculty of Medicine Université de Montréal Montreal Quebec Canada

**Keywords:** myotonic dystrophy Type 1, senescence, senolytics, senomorphics

## Abstract

Myotonic dystrophy type 1 (DM1) is considered a progeroid disease (i.e., causing premature aging). This hypervariable disease affects multiple systems, such as the musculoskeletal, central nervous, gastrointestinal, and others. Despite advances in understanding the underlying pathogenic mechanism of DM1, numerous gaps persist in our understanding, hindering elucidation of the heterogeneity and severity of its symptoms. Accumulating evidence indicates that the toxic intracellular RNA accumulation associated with DM1 triggers cellular senescence. These cells are in a state of irreversible cell cycle arrest and secrete a cocktail of cytokines, referred to as a senescence‐associated secretory phenotype (SASP), that can have harmful effects on neighboring cells and more broadly. We hypothesize that cellular senescence contributes to the pathophysiology of DM1, and clearance of senescent cells is a promising therapeutic approach for DM1. We will discuss the therapeutic potential of different senotherapeutic drugs, especially senolytics that eliminate senescent cells, and senomorphics that reduce SASP expression.

## Graphical abstract

Cellular senescence accumulates in the skeletal muscle of patients with myotonic dystrophy Type 1, leading to the production of senescence‐associated secretory phenotype (SASP) factors that have detrimental effects. Targeting these senescent cells with senotherapeutics could restore muscle homeostasis and slow disease progression.

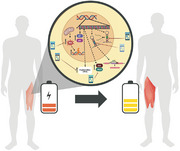

**Abbreviations**
ASO: antisense oligonucleotides
CAR: chimeric antigen receptor
CELF1: CUGBP Elav‐Like Family Member 1
DM1: myotonic dystrophy type 1
DMPK: dystrophia myotonica protein kinase
EMIQ: enzymatically modified isoquercetin
FAP: fibroadipogenic progenitor
HSC: hematopoietic stem cell
HUVEC: human umbilical vein endothelial cell
IMR90: human diploid fibroblasts
MBNL: Muscleblind‐like
SASP: senescence‐associated secretory phenotype

## 1 Introduction

Myotonic dystrophy type 1 (DM1) is the most common form of muscular dystrophy in adults. It has a global prevalence of 1 in 8000 [1], but its prevalence is much higher in some regions reaching 1 in 630 in the Saguenay–Lac‐St‐Jean region in Québec (Canada) [2], and the mutation responsible for DM1 has been found at 1 in 2100 in New York state (United States of America) [3]. DM1 is caused by a CTG expansion in the 3′ untranslated region of the *dystrophia myotonica protein kinase (DMPK)* gene [4, 5, 6]. The expanded RNA of the *DMPK* gene containing the CUG repeats traps splicing regulators such as Muscleblind‐like (MBNL) in the nucleus, thus preventing them from regulating alternative splicing and resulting in aberrant alternative splicing. Another splicing regulator, CUGBP Elav‐Like Family Member 1 (CELF1), is phosphorylated in DM1 which leads to its hyperactivation and thus also participates in the misregulation of alternative splicing [7, 8]. Other RNA‐binding proteins such as Staufen1 are also altered in DM1 [9]. *DMPK* expanded RNA was shown to affect transcription factors such as SP1, which will affect gene expression [10]. Many aspects of gene expression such as alternative polyadenylation, microRNA levels, and RNA localization have been shown to be altered in DM1 [7, 11–14]. Overall, the CTG expansion mutation in *DMPK* triggers a cascade of events leading to RNA toxicity.

**FIGURE 1:**
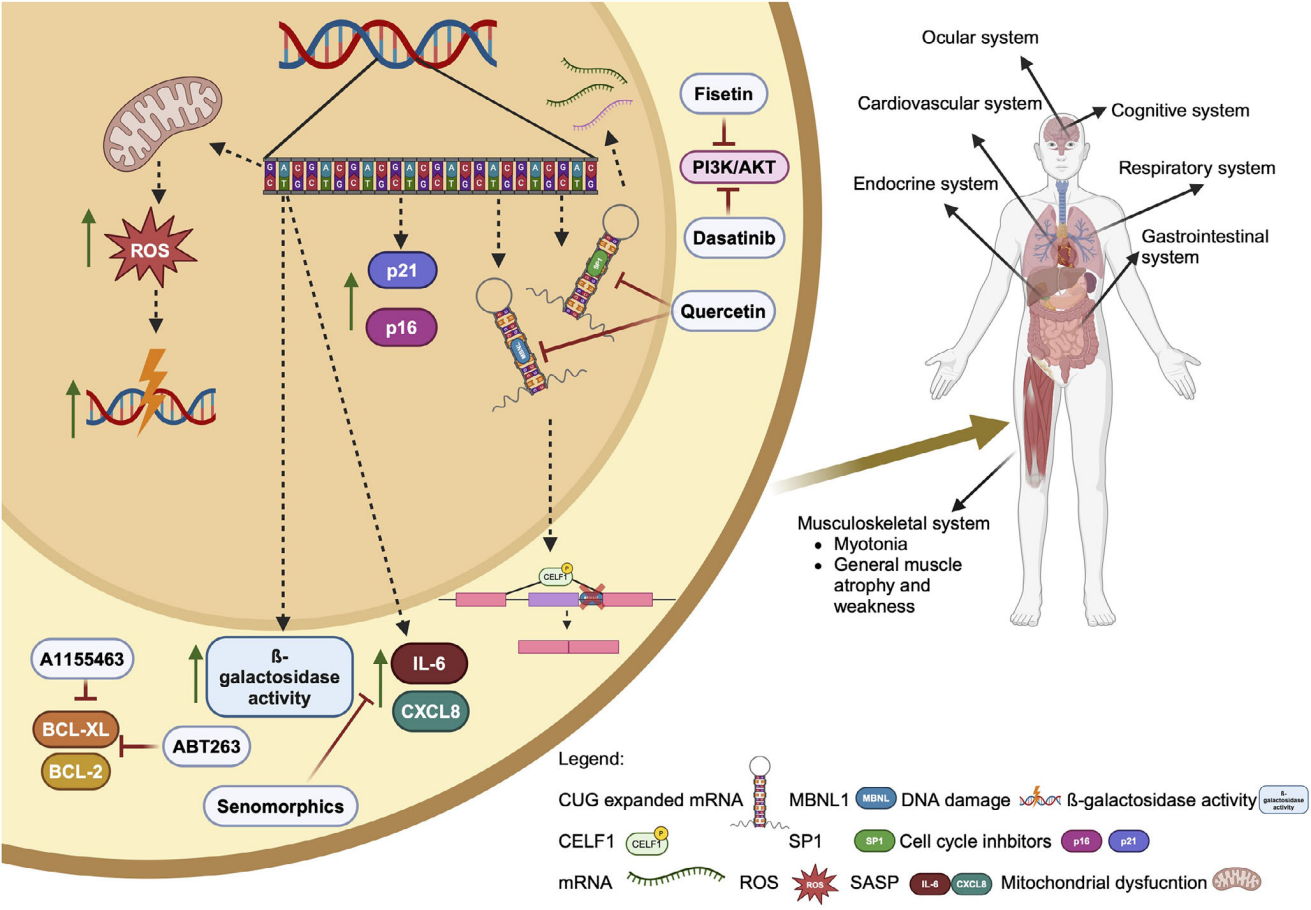
DM1, senescence, and senotherapeutics. Representation of the main pathophysiological mechanism and clinical manifestations in DM1. DM1 is caused by a CTG repeat in the *DMPK* gene. The RNA traps MBNL proteins to the nucleus which results in alterations in alternative splicing. It also traps transcription factors such as SP1, which results in gene dysregulation. This also results in mitochondrial dysfunction and increase in ROS which leads to damage to DNA. An increase in the expression of cell cycle inhibitors (p16 and p21) as well as SA‐ß‐galactosidase activity and SASP (e.g., IL‐6 and CXCL8) production is also observed. The effect of different senolytics and senomorphics and their mechanisms of action to target hallmarks of cellular senescence in DM1 is also represented. Created with BioRender.com. DM1, myotonic dystrophy type 1; DMPK, dystrophia myotonica protein kinase; MBNL, Muscleblind‐like; ROS, reactive oxygen species; SASP, senescence‐associated secretory phenotype.

DM1 can be divided into five clinical phenotypes based on the age at onset of the disease: congenital (less than 1 month), infantile (between 1 month and 10 years), juvenile (between 10 and 20 years), adult (between 20 and 40 years), and late‐onset (more than 40 years) [15]. DM1 is a multisystemic disease and patients can present various clinical manifestations including cognitive impairments, cataracts, respiratory insufficiency, gastrointestinal problems, heart problems, diabetes, muscular weakness, and myotonia [16]. Despite the identification of the main pathophysiological mechanism, there is only a moderate correlation between the length of CTG repeat expansion and clinical manifestations [17]. A summary of the clinical manifestations in DM1 as well as the physiopathological mechanisms are shown in Figure 1. There is a significant heterogeneity in the presence and severity of clinical manifestation and the rate of progression of the disease between the sexes and the different clinical phenotypes [15, 16, 18–22].

DM1 presents clinical symptoms (e.g., muscle weakness, cognitive impairments, and cataracts) as well as molecular features (e.g., mitochondrial dysfunction, nuclear envelope abnormalities, and cellular senescence) associated with progeroid syndrome (premature aging) [19, 23, 24]. Among these changes, cellular senescence has gained significant interest in the field of aging. Senescence is a state in which cells are irreversibly removed from the cell cycle. Although there is not a universal marker of cellular senescence, these cells acquire different specificities [25]. They have a distinct appearance as they are flatter and larger than nonsenescent cells. They express senescence‐associated beta‐galactosidase activity, which indirectly reflects an increase in lysosomal content and residual activity at suboptimal pH. They can also show signs of nuclear envelope disturbance and DNA damage. They usually upregulate the expression of cyclin‐dependent kinase inhibitors such as p16, p21, and/or p53, and they do not express proliferation markers such as Ki67 expression or 5‐bromodeoxyuridine incorporation [26]. They also secrete a cocktail of cytokines (e.g., interleukin‐6), chemokines (e.g., CXCL8), growth factors (e.g., growth differentiation factor 15), and proteases (e.g., metalloproteinase‐1), called the senescence‐associated secretory phenotype (SASP) [27, 28]. The production of SASP is highly variable depending on the type of senescent cells and the inducer [28]. Transient exposure to SASP could play an important role to regulate tissue regeneration [29, 30]; however, its prolonged expression leads to detrimental effects on neighboring cells and on the whole organism [31]. For instance, the transplantation of senescent preadipocytes intraperitoneally led to muscle weakness and reduced physical activity in mice [32]. Secretion of SASP can also trigger senescence of neighboring cells, a process called secondary senescence, thereby promoting a vicious cycle [33].

## 2 Hypothesis

With DM1 sharing many similarities with premature aging, we propose that cellular senescence is a hallmark of the disease, which contributes to the disease severity and/or progression. We hypothesize that senotherapeutics are effective in eliminating defective cells in DM1 and/or reduce their expression of SASP to restore a healthy systemic environment. Further, we hypothesize that senotherapeutics have a multisystemic effect on different organs affected in DM1. Finally, we propose that senolytics and senomorphics could be used in combination with other therapies to optimize the beneficial effect on the patient's symptoms.

## 3 Signs of Cellular Senescence in DM1

Accumulating evidence indicates that cellular senescence is a hallmark of DM1 (Table 1). Cells collected from DM1 patients demonstrate signs of senescence, which are summarized in Figure 1. Myoblasts isolated from a skeletal muscle of congenital DM1 fetuses [34], and from the *tibialis anterior* (TA) [35], *biceps brachii* [36], and *vastus lateralis* [37] of DM1 patients, all presented reduced growth rate in comparison to controls. Similar findings were obtained with primary culture of DM1 fibroblasts [38] and with an inducible CUG repeat model of human lung fibroblast cell line (TIG‐3 cells) [39]. Histological analyses revealed that myoblasts from DM1 skeletal muscle (*biceps brachii*), as well as DM1 fibroblasts showed the classical morphology of senescent cells (larger and flatter) [34, 36, 38]. DM1 fibroblasts also have bigger nuclei that are not shaped correctly, as well as mislocalization of the nuclear envelope proteins emerin, lamin A/C, and B1 [40]. Myoblasts with lower expression of lamin A also had a higher number/percentage of nuclei with invaginations, similar to what is seen in aging or cancer cells, as well as no expression of Ki‐67 [24]. Other senescence markers such as senescence‐associated β‐galactosidase activity were also increased in myoblasts from DM1 skeletal muscle (*biceps brachii* and *vastus lateralis*) as well as DM1 fibroblasts and inducible CUG repeat model from TIG‐3 fibroblast cells [34, 36–39, 41]. Thus, many markers of senescence have been identified in DM1.

**TABLE 1:** Summary of cellular and molecular characteristics of senescence in myotonic dystrophy type 1 (DM1) models.

Models	Characteristics	References
DM1 fibroblasts	↓ Growth rate, classical morphology of senescent cells, ↑ ß‐galactosidase activity, ↑ *P16,P14,P27,P21*, ↓ *BMI1*, ↓ cell cycle, cell division and DNA replication and repair pathways, ↑*IL‐6, TNF‐α* and *CCL5*	[38]
DM1 fibroblasts	Larger nuclei, mislocalization of emerin, lamin A/C, and B1	[40]
Myoblasts isolated from skeletal muscle of DM1 congenital fetuses	↓ Growth rate, classical morphology of senescent cells,↑ ß‐galactosidase activity	[34]
DM1 myoblasts	↓ Lamin A/B/C ↑ % of nuclei with invagination and no Ki‐67	[24]
Myoblasts isolated from DM1 *tibialis anterior*	↓ Growth rate	[35]
Myoblasts isolated from DM1 *biceps brachii*	↓ Growth rate, classical morphology of senescent cells	[36]
Myoblasts isolated from DM1 *vastus lateralis*	↓ Growth rate, ↑ ß‐galactosidase activity, ↑ *P16*, *P21*, ↓ cell cycle, muscle structure development pathways, ↑ cytokine signaling, cellular response to stress, ROS production, apoptosis, and senescence pathways, ↑ *CXCL1*, *CXCL8*, *IL6*, and *MMP1*	[37]
Myoblasts isolated from different proximal limb muscles	↑ *P16, P14, P27*, ↓ *BMI1*,↓ cell cycle, cell division, and DNA replication and repair pathways	[38]
Congenital DM1 myoblasts	↑ *IL‐6*	[43]
Inducible CUG repeat (human fibroblast cell line)	↓ Growth rate, ↓ BrDU incorporation, ↑ ß‐galactosidase activity, ↑p53, p16, p21, ↑ ROS production, ↑ DNA damage	[46]
Inducible CUG expansion C2C12	↑ *IL‐6*	[43]
DM1 human muscle	↑ Satellite cells expressing p16, ↑ P16, P21 expression	[37]
DM1 *Drosophila*	↑ *Dacapo* ↓ *Psc*	[38]
DMSXL mouse	↑ *P19, P27, P21*, ↓ *Bim1, Ki67* ↓ cell cycle, cell division, and DNA replication and repair pathways	[38]
HSA^LR^ mouse	↑ *Tmem158*, *Cdkn1a* and *Cdkn2a*,	[42]

The expression of cell cycle regulators that have been associated with senescence are also changed in DM1 cells. An upregulation of the cell cycle inhibitors *p16*, *p14*, *p27*, and *p21*, and a downregulation of the proto‐oncogene *BMI1* have been shown in DM1 fibroblasts compared to controls [38]. Similar findings were observed in the TA muscle of DMSXL mouse, in DM1 myoblasts, and in peripheral blood mononuclear cells (PBMCs) from DM1 patients [38]. Moreover, *Dacapo*, an homolog of *p21* and *p27*, was upregulated in a *Drosophila* model of DM1 compared to controls, whereas *Psc* which is the homolog of *BIM1* was downregulated [38]. Myoblasts from DM1 patients had more elevated *P16* expression than myoblasts from controls [37]. In situ muscle sections from DM1 patients showed a higher proportion of satellite cells (muscle stem cells) expressing *P16*, and they also had higher expression levels of *P16* and *P21* compared to controls [37]. In gastrocnemius muscle of the HSA^LR^ mouse model, *Tmem158*, *Cdkn1a*, and *Cdkn2a* were upregulated compared to controls [42]. Therefore, cell cycle inhibitors are upregulated in DM1, which is also an indicator of senescence.

Transcriptomics analysis revealed that several pathways related to senescence are dysregulated in DM1 [37, 38]. Cell cycle, cell division, and DNA replication and repair pathways were downregulated in DM1 fibroblasts. In PBMCs and myoblasts derived from DM1 patients and TA from DMSXL mice, biological processes related to cell cycle, cell division, and DNA replication and repair were downregulated [38]. Similarly, in myoblasts from DM1 patients, genes involved in cell cycle and muscle structure development were downregulated, whereas genes implicated in cytokine signaling, cellular response to stress, reactive oxygen species (ROS) production, apoptosis, and senescence were upregulated [37]. In TIG‐3 fibroblast cells containing CUG repeat expansion, there was an upregulation of genes implicated in the p53 signaling pathway and genes implicated in cell cycle and a downregulation in genes involved in the response to oxidative stress [39]. These transcriptomic changes are associated with the molecular signature of cellular senescence.

The expression of SASP is also upregulated in DM1 models. Single‐cell RNAseq of DM1 myoblasts and age‐ and sex‐matched healthy controls showed an upregulation of inflammatory cytokines/chemokines such as *CXCL1*, *CXCL8*, *IL6*, and proteases like *MMP1* [37]. Comparison to other published senescent datasets showed that this molecular signature was associated with SASP [37]. Similarly, an upregulation of *IL‐6*, *TNFa*, and *CCL5* has been shown in DM1 fibroblasts compared to controls [38]. There was an upregulation of *IL‐6* in PBMCs from DM1 patients [38]. Inducible CUG expansion in the C2C12 myoblast model also showed that toxic RNA induces IL‐6 upregulation [43]. IL‐6 expression was shown to be upregulated in congenital DM1 muscles and correlated with muscle immaturity [43]. Moreover, a negative correlation between IL‐6 serum level and muscle strength (upper limb and lower limb) and physical function was observed in a cohort of DM1 adults [37]. Therefore, components of the SASP, which are another canonical marker of senescence, have also been found in various models of DM1.

Altogether, these findings indicate that the main characteristics of senescence, that is, cell cycle arrest, nuclear envelope abnormalities, lysosomal content, and SASP, are observed in DM1. Moreover, some of these markers are correlated with disease severity suggesting that targeting cellular senescence/SASP has therapeutic potential for the treatment of DM1.

Noteworthy, there is emerging evidence suggesting that cellular senescence is also observed in myotonic dystrophy Type 2 (DM2). Myoblasts cultured from satellite cells collected from the *biceps brachii* of DM2 patients showed senescence‐like ultrastructural changes such as increased cytoplasmic vacuoles and accumulation of heterochromatin [44]. An increased nuclear invagination was also observed in DM1 and DM2, which was associated with a reduction in the expression of lamin subtypes A, B, and C, but only in DM1 [24]. Another study showed that DM2 myoblasts have lower proliferation rate and cell division capacity than control [36]. However, in contrast to DM1 myoblasts, this reduction was not associated with an increase in p16 expression, but rather with a reduction in telomere length. It was also shown in another study that CCUG repeat triggers a cascade that affects protein turnover in DM2, leading to the stabilization of protein such as p21 that are less degraded by the proteasome system [45]. These results suggest that senescence is a common process in DM1 and DM2, but the underlying mechanism might differ. There is a need for further studies on the fundamental processes regulating cellular senescence in DM2.

## 4 Underlying Mechanism Regulating Cellular Senescence in DM1

Cellular senescence can be triggered by a wide variety of environmental stresses or intrinsic dysfunctions (e.g., DNA damage, telomere shortening, high levels of ROS, and stimulation of oncogenes). Reduced telomere length has been shown to trigger senescence, but this mechanism does not appear to be implicated in DM1. Indeed, telomere length in satellite cells from TA muscle from DM1 patients [35], satellite cells from muscle from DM1 fetuses [34] as well as DM1 myoblasts [36] were longer than controls when they reached replicative senescence. Another study by Hasuike et al. did not see a difference in telomere length between their inducible expanded CUG repeat model and the control cells when they stopped growing in vitro [46]. These results might be tissue‐dependent as faster telomere abrasion has been observed in the blood of DM1 patients compared to controls, although these results have not yet been confirmed in a replication cohort [47]. Altogether, these results do not seem to indicate that telomere shortening is a main driver for senescence in DM1.

One potential mechanism is that the accumulation of toxic RNA triggers cellular senescence. In support of this hypothesis, DM1 fibroblasts expressing abundant nuclear foci are more prone to have abnormal nuclear envelope organization [40]. Similarly, the number of RNA foci was higher in senescent myogenic cells than in nonsenescent cells. Moreover, senescent cells also expressed higher levels of ROS [37]. This increased ROS expression has also been observed in fibroblasts from DM1 patients [38]. DM1 patients also had less glutathione peroxidase, glutathione‐S‐transferase and reduced glutathione in their blood [48]. DM1 cell models containing CTG repeat expansion were more sensitive to oxidative stress induced by smaller doses of methylmercury [49]. Noteworthy, chronic ROS production has been shown to induce DNA damage and senescence [50]. Accordingly, using the TIG‐3 cell model it was shown that the induction of the expanded CUG RNA provokes an increase in mitochondrial ROS production and DNA damage [39]. Thus, ROS levels are elevated in DM1, and this could contribute to senescence by inducing DNA damage.

The activation of cell cycle checkpoints plays a key role in the DNA damage response [51]. An increase in the expression of p16 has been observed in DM1 cells [37, 38]. The overexpression of CDK4 to bypass p16 expression in DM1 myoblasts increased the number of cell divisions to a similar level than control cells [34]. Altogether, chronic DNA damage can result in the overexpression of cell cycle inhibitors in DM1 that are known to trigger senescence.

The accumulation of RNA toxicity could also activate inflammatory response pathways. Using a C2C12 myoblast cell line model carrying an 800 CTG repeat expansion driven by Cre excision of a transcription terminator cassette, it was shown that RNA toxicity stimulates the NF‐kB pathway and the expression of downstream inflammatory genes, especially IL‐6 [43].

Anti‐senescence mechanisms, such as autophagy, are also downregulated in DM1. Following 24 h of fasting, HSA^LR^ mice had less activation of the AMPK pathway, lower autophagy induction, and less degradation than controls. DM1 myotubes also displayed reduced autophagy induction following starvation [52]. DM1 patients also showed lower autophagy than controls [53]. These findings suggest that the mechanisms to prevent or remove senescent cells are less efficient in DM1.

Altogether, these findings suggest that the accumulation of RNA toxicity triggers mitochondrial dysfunction and ROS production, which induces DNA damage, cell cycle arrest, and SASP production. Moreover, the lack of efficient antisenescence mechanisms reduced the ability to remove these senescent cells.

## 5 Types of Senotherapeutics

Senotherapeutics can be divided into different classes, especially senomorphics (or senostatics) and senolytics (Figure 1). Senolytics are drugs that are more specific in eliminating senescent cells than nonsenescent cells. These drugs have been developed to inhibit different pathways that maintain the survival of senescence cells and block their entry into apoptosis. Senolytics target pathways such as PI3K/Akt, BCL, FOXO4‐p53, HSP90, and others [54]. Dasatinib and quercetin are the first senolytics that were identified [55], followed by other first‐generation senolytics such as fisetin and navitoclax (ABT263) [56, 57]. This field is rapidly expanding and second‐generation senolytics, such as lysosomal and SA‐β‐Gal activated prodrugs, are being identified using different methods such as high throughput screening [58].

The underlying mechanisms regulating cellular senescence are complex processes that are affected by many factors such as stress inducer and cell type. Accordingly, the efficacy of senolytics will vary depending on the conditions. For instance, dasatinib killed senescent human preadipocytes, and to a lesser degree senescent human umbilical vein endothelial cells (HUVECs) [55]. On the contrary, quercetin was more efficient in killing senescent HUVEC than preadipocytes. If both dasatinib and quercetin were combined, they were able to kill both senescent preadipocytes and HUVEC. Another senolytics, navitoclax (ABT263), targeting the BCL‐2/BCL‐XL pathway eliminate senescent HUVEC and human diploid fibroblasts (IMR90) but not senescent human preadipocytes [57]. Inhibitors of BCL‐XL, such as A1155463 and A1331852, also showed senolytic effect in senescent HUVEC and IMR90 but not preadipocytes [56]. FOXO4‐DRI is a peptide that has been designed to inhibit the interaction between p53 and FOXO4. It kills IMR90 senescent cells, with more specificity toward senescent cells compared to quercetin and dasatinib, and at lower doses than ABT‐737 [59]. Fisetin is another senolytic targeting the PI3K/AKT pathway senolytics, which kills senescent HUVEC and senescent mouse embryonic fibroblast (MEF) [60], but not IMR90 or human preadipocyte by inducing apoptosis [56]. Using single‐cell RNAseq from senescent and nonsenescent fibroadipogenic progenitors (FAPs) and satellite cells collected from muscle, it was shown that CRYAB is a strong senescence‐induced gene, which can be targeted by 25‐hydroxycholesterol to inhibit its aggregation. This senolytic targets multiple senescent cell types such as fibro‐adipogenic progenitors, satellite cells, mouse dermal fibroblasts, IMR‐90, human cardiac microvascular endothelial cells, human liver stellate cells, human renal proximal tubule epithelial cells, and human articular chondrocytes [61]. This nonexhaustive list of senolytics highlights that the specificity and efficacy of these drugs vary considerably depending on the condition.

One of the main challenges associated with the use of senolytics is their off‐target effects. Different approaches have been developed to reduce these side effects. For instance, Muñoz‐Espín et al. found a way to deliver drugs directly to senescent cells by encapsulating them in galacto‐oligosaccharide [62]. The combination of navitoclax with an acetylated galactose group was more specific to senescent cells and reduced the navitoclax‐driven thrombocytopenia in mice [63]. Another group generated an intracellular oligomerization system that disrupted the mitochondrial membrane of senescent cells, which have weaker integrity than healthy cells [64]. Different antibody‐based cytotoxicity approaches were also tested to improve specificity. Particularly, chimeric antigen receptor (CAR) T cells targeting the urokinase‐type plasminogen activator receptor on senescent cells were shown to efficiently ablate these cells [65, 66]. These different methods directly target senescent cells, which should reduce side effects.

Senomorphics are another class of senotherapeutics that have gained significant interest in the field. These immunomodulators reduce SASP expression without killing the senescent cells. These drugs can target pathways such as NF‐kB, p38MAPK, JAK/STAT, ATM (ataxia‐telangiectasia mutated) protein kinase, and others. Rapamycin is a senomorphics that has shown a potent capacity to suppress SASP in a variety of models; however, side effects such as metabolic defects, thrombocytopenia, and hyperlipidemia are a concern [67]. Metformin is another senomorphic that can target multiple pathways such as NF‐kB and AMPK, and reduce SASP expression in different cell types [67]. JAK/STAT is another pathway, that is, upregulated in senescent cells, which can be targeted by inhibitors such as ruxolitinib. JAK inhibitors reduced the expression of SASP (e.g., IL‐6, IL‐8, and CXCL1) in senescent human primary preadipocytes and HUVEC [68].

Other approaches targeting senescence are currently emerging. For instance, senoblockers are being developed to prevent the appearance of senescence cells, rather than to induce their removal [69]. Alternatively, senoreversers, such as reversin, were shown to restore some of the features of pre‐senescent myoblasts (e.g., reduce SA‐β‐Gal expression and γH2AX levels, increase autophagy, and restore metabolism) without rescuing completely the proliferative potential of these cells [70].

Overall, there is a rapidly expanding number of senotherapeutic approaches. Considering that the underlying mechanism of senescence and SASP vary substantially depending on the disease, tissue, and condition, it is crucial to develop personalized medicine approaches to transfer these treatments in the DM1 field.

## 6 Senolytics and Human Conditions

The studies using senolytics in other neuromuscular conditions bring important information to the DM1 field, especially with regard to advanced aging since DM1 displays characteristics of a progeroid syndrome. A pioneer study showed that the negative impact of senescent cells on lifespan in naturally aged mice could be reverted by AP20187, a dimerizer inducing FKBP‐Caspase‐8 fusion [71]. Different models of premature aging have also been used to study the impact of senolytics. Treatment of irradiated C57BL/6 mice with ABT263 killed senescent hematopoietic stem cells (HSCs), without decreasing the number of HSCs or hematopoietic progenitor cells [72]. It also lessened the myeloid skewing of HSCs, the number of HSCs in quiescence and diminished the number of HSCs with DNA damage which are all markers of aging of HSCs. Similar findings on the rejuvenation of HSCs and the reduction of the number of senescent muscular stem cells were also observed in naturally aged mice [72]. In another accelerated aging model, the Xpd^TTD/TTD^ mice, which accumulate high levels of DNA damage, FOXO4‐DRI treatment reduces senescence markers, mitigates loss of renal function, and improves their mobility. Similar findings were observed in naturally aged p16:3MR mice treatment with FOXO4‐DRI [59]. In another progeroid syndrome model, the Ercc1^−/Δ^ mice that have impaired DNA repair mechanism, the treatment with the HSP90 inhibitor 17‐DMAG, reduced senescence markers (e.g., p16 expression, IL‐6 levels), diminished the symptoms associated with age, and increased lifespan [73]. Similarly, treatment of fisetin in Ercc1^−/Δ^ mice reduced senescence, SASP markers, and oxidative stress [60]. Naturally aged mice treated with fisetin also had less senescent cells and longer lifespan [60]. The combination of dasatinib and quercetin also showed the ability to reduce senescence in old animals [55], increase muscle growth and regeneration, improve physical function [74, 75], improve metabolic function [76], and reduce learning and memory problems [77]. CAR T cells targeting the urokinase plasminogen activator receptor‐positive senescence cells were shown to be safe and increased exercise capacity and metabolic function in physiologically aged mice [66]. In addition to senolytics, the administration of senomorphics such as ruxolitinib (JAK inhibitor) to old mice reduced systemic inflammation markers and enhanced physical function [68]. Altogether, these findings indicate that different types of senotherapeutics have demonstrated therapeutic effects in different tissues/organs in various models of aging, which is relevant for DM1.

Senolytics have also been used for the treatment of other genetic myopathies, such as Duchenne muscular dystrophy (DMD). This disease is associated with increased signs of cellular senescence over time in their muscle stem cell population [78]. Treatment of DMD rats with ABT263 reduced p16, p19, and p21 expression. Treated rats did not lose body weight or muscle strength during treatment, while vehicle‐treated rats did. They also had more regenerating myofibers and lower expression of SASP (e.g., IL‐6, TGF‐𝛃, and IL‐1𝛃) [79]. Treatment of *mdx*‐utrophin double knockout mouse model with fisetin reduced the number of senescent immune cells and their expression of SASP, increased the muscle stem cell pool, and diminished fibrosis in skeletal muscle [80]. These findings indicate that senolytics ameliorate the phenotype in different DMD animal models.

Senolytic treatment has also been applied in other neuromuscular diseases such as multiple sclerosis, amyotrophic lateral sclerosis (ALS), and Friedreich's ataxia. In an autoimmune encephalomyelitis mouse model of multiple sclerosis, ABT263 treatment reduced motor impairments and white matter inflammation as well as improved visual function [81]. Treatment with ABT263 in the G93A mouse model of ALS did not diminish weight loss or disease progression, nor did it affect senescence or SASP makers. However, in contrast to ABT263, treatment of G93A fibroblasts with dasatinib and quercetin significantly lowered *p16* and *p21* expression [82]. In Friedreich's ataxia mouse model (Fxn‐/‐mouse) that was exposed to hypoxia, treatment with ABT263 lessened vascular disease [83]. Thus, senolytics seem to be a promising avenue for the treatment of neuromuscular diseases.

Considering their high therapeutic potential, there has been an increasing number of clinical trials using senolytics for different conditions, such as cancers, Alzheimer's disease, or idiopathic pulmonary fibrosis (no results of trials on neuromuscular diseases up to now) [84]. Most of these early‐phase trials are ongoing and aim to evaluate tolerability and safety. A study on nine patients with chronic obstructive pulmonary disease treated either with placebo or quercetin (500, 1000, or 2000 mg) for 7 days did not observe serious adverse events or changes in safety parameters [85]. In another study, dasatinib (10 mg) and quercetin (1000 mg) were given to five Alzheimer's patients for 2 days followed by 14 days without any treatment, which was repeated five times. No severe adverse effects or differences in safety parameters were reported except for a higher total cholesterol value after treatment [86]. A 3‐day treatment of dasatinib (100 mg) and quercetin (1000 mg) was given to nine diabetic kidney disease patients and was well tolerated as no serious adverse events happened. The treatment reduced senescence markers in adipose tissue, but not in the skin. It also diminished the blood expression of SASP, such as IL‐6, IL1a, and MMP‐9 and ‐12 [87]. In another clinical trial, dasatinib (100 mg) and quercetin (1250 mg) were given to 14 idiopathic pulmonary fibrosis patients 3 days per week for 3 weeks [88]. There were no changes in safety parameters, but one serious adverse event happened after the treatment. There was no change in pulmonary function in the patients following treatment, but they showed clinical and significant improvement in physical tests, such as 6‐min walk distance and 4‐m gait speed. No changes in the levels of SASP were observed following treatment, but a correlation between SASP and function were noted [88]. Another study on idiopathic pulmonary fibrosis came to a different conclusion. In this study, 12 patients were given a placebo or dasatinib (100 mg) and quercetin (1250 mg) for 3 days per week for 3 weeks. No serious adverse events were detected, but the treatment did not improve frailty, pulmonary, or physical function [89]. Overall, these pioneering studies generally demonstrate that senolytics are well tolerated and hold some therapeutic potential, but further studies are needed to evaluate their efficiency in reducing senescence and improving functional outcome in different human diseases.

## 7 Senolytics in DM1

Different groups working on DM1 used various models to test the therapeutic potential of senolytics for the treatment of DM1. Quercetin, navitolax, or dasatinib treatment in DM1 and control fibroblasts reduced the number of cells with SA‐β‐galactosidase activity [38]. Quercetin and dasatinib treatment also reduced *IL6* expression. An increase in the number of cell positive for Caspase 3 is also observed suggesting that these senolytic drugs promote the apoptosis of senescent cells. Treatment with quercetin in a *Drosophila* DM1 model partially restores the levels of cell cycle genes such as *String* (*Cdc45*), *Dacapo (p21/p27)*, and *Psc* (*Bmi1*). The treated *Drosophila* moved more and lived longer than the untreated ones [38]. In another model, DM1 myoblasts or healthy aged‐ and sex‐matched controls were treated with six different senolytics (FOXO4‐DRI‐peptide, fisetin, dasatinib + quercetin, navtoclax, A1331852, and A1155463). Drugs such as navitoclax and fisetin showed the capacity to eliminate cells only in DM1 samples and not in healthy controls at specific concentrations, but the most efficient and specific candidate was A1155463, a BCL‐XL inhibitor. Treatment with A1155463 reduced senescence markers in DM1 myoblasts (e.g., number of SA‐β‐galactosidase positive cells, *P16* expression) as well as SASP expression (e.g., reduced levels of *CSF3*, *CXCL1*, *CXCL8*, *CCL2*, *MMP‐1*, and *MMP‐3*) [37]. The treatment also augmented DM1 myoblast proliferation (KI67) and differentiation markers (MYOG) in vitro, as well as cell engraftment in vivo [37]. Another study using the DM1 HeLa cell model to screen natural products identified quercetin as a selective modulator of RNA toxicity [90]. Quercetin reduced the expression of r(CUG)480 expansion RNA levels potentially through binding the CTG repeats. Treatment with quercetin in DM1 fibroblasts reduced *DMPK* expression and rescued splicing of *INSR* exon 11 and *FNLB* exon 31 without affecting splicing in control fibroblasts. Treatment of DM2 fibroblasts also rescued splicing of *INSR* exon 11 and *FNLB* exon 31 as well as reducing *CNBP* expression. Dasatinib, A1155463, and quercetin treatment reduced the P16 level, but only quercetin was able to rescue splicing and reduce *DMPK* expression. Treatment of DM1 myotubes rescued splicing of *MBNL1* exon 5 and *SYNE1* exon 137 as well as reducing *DMPK* without affecting splicing in controls. High‐dose treatment of enzymatically modified isoquercetin (EMIQ) for 6 or 12 weeks in HSA^LR^ mice was able to diminish HSA CUG transgene expression and rescue splicing of *Clcn1* exon7 and *Atp2a1* exon 22. Many other splicing events were rescued in the HSA^LR^ mice while only modifying the expression of 2% of genes. Myotonia was also improved with 6‐ and 12‐week treatments of EMIQ in HSA^LR^ mice [90]. Altogether, these findings highlight the therapeutic potential of senolytics for DM1.

## 8 Senolytics Combination With Other Therapies

Senolytic treatments in DM1 have limitations, as most of these compounds do not directly target the root cause of the disease, that is, the toxic accumulation of expanded CUG messenger RNA. As such, an interesting idea will be to use one or more senolytics in combination with other treatments for improved activity. One example of a combination that has been used in clinical trials for the treatment of other diseases is the combination of two senolytics, dasatinib and quercetin. This combination seems to be well tolerated in clinical trials, but further studies will be needed to evaluate its efficiency and ensure that no adverse events are associated with this combination, especially if these treatments are used for a longer period than past trials [85, 86, 87, 88]. As mentioned previously, the combination of those two senolytics kill different types of senescent cells (e.g., both senescent HUVEC and preadipocytes) [55]. Therefore, combining different drugs could help to target senescence in different organs, which would be advantageous for multisystemic diseases such as DM1. Another advantage of combining senolytics treatments is that it is possible to achieve the same efficiency with lower doses of each senolytics, which could help reduce the adverse effects. The combination of both ABT‐263 and piperlongumine was also a more efficient senolytic compared to when they were used separately in WI‐38 cells [91]. The combination of both EF24 and ABT263 was more efficient to kill senescent WI‐38 cells than each of them separately [92].

It is also possible to combine senolytics with another type of treatment to improve their efficiency. Combining navitoclax with pan‐mTOR inhibitors (which are not senolytics themselves) reduces the doses of navitoclax needed to kill senescent NIH3T3 or HUVEC cells. It also further improved the lifespan of a premature aging model of *Drosophila* compared to treatment with navitoclax alone [93]. Combining senolytics administered as a “hit and run” treatment to eliminate the senescent cells, followed by senomorphics treatment to delay the return of the senescent cells could also be an interesting approach to explore. Another interesting avenue is to combine senolytics treatment with exercise which has also been identified to hold senolytic potential itself [94]. Furthermore, exercise was demonstrated to be beneficial in DM1 patients. Men who underwent a 12‐week lower limb strength training program reduced their fatigue, sleepiness, and apathy while improving their lower limb muscle strength, walking speed and 30‐s sit‐to‐stand score [95, 96]. Women improved their knee and hip extensor strength, apathy, depression, pain interference, and lower limb function [97]. At the molecular level, training also rescued alternative splicing and differential gene expression [98], and modulated protein expression in men [99]. Mitochondrial function was also improved in men by the strength training program [100]. Senolytics could be combined with strategies that directly target CUG‐expanded mRNA, such as antisense oligonucleotides (ASO). Considering the challenges in delivery of ASOs in skeletal muscle so far, senolytics could be a way to eliminate the defective cells that are not well targeted with ASOs, which could have a combinatory effect [101]. Altogether, there are several advantages to the combination of senolytics with other therapies.

## 9 Conclusion

There are several signs of senescence in DM1, including the presence of SASP. Accumulating evidence indicates that these features are associated with disease severity. As such, senolytics removing senescent cells or senomorphics targeting the SASP are promising therapies for this disease, either on their own or in combination with other therapeutics strategies including exercise or ASOs to improve their efficiency. However, there are still many unknowns in this emerging field, such as the impact of senescence in different tissues in DM1, and the potential of senotherapies to target the multisystemic symptoms of the disease. The current review highlights the need for more studies to evaluate the potential of senotherapeutics in DM1.

## Author Contributions

**Cécilia Légaré**: reviewed the literature and wrote the manuscript. **J Andrew Berglund, Elise Duchesne, and Nicolas A. Dumont**: wrote and revised the manuscript.

## Conflicts of Interest

J.A.B. serves on the Scientific Advisory Committee for the Myotonic Dystrophy Foundation, has consulted or currently consults for Entrada Therapeutics, Juvena Therapeutics, Kate Therapeutics, D.E. Shaw Research, Dyne Therapeutics, Syros Pharmaceuticals, and Wayfinder Biosciences, and has received research funding from Agios Pharmaceuticals, Biomarin Pharmaceuticals, PepGen, Syros Pharmaceuticals, and Vertex Pharmaceuticals. J.A.B. has received licensing royalties from the University of Florida and holds patents and patent applications at the University of Albany related to the subject matter. J.A.B. also is a funder and has a financial interest in Repeat RNA Therapeutics Inc. E.D. has consulted or currently consults for Dyne Therapeutics, PepGen, Avidity, and Vertex Pharmaceuticals, and has received research funding from Vertex Pharmaceuticals.

## Data Availability

Data sharing not applicable to this article as no datasets were generated or analyzed during the current study.
